# Chiral trimethylsilylated *C*_2_-symmetrical diamines as phosphorous derivatizing agents for the determination of the enantiomeric excess of chiral alcohols by ^1^H NMR

**DOI:** 10.1186/1860-5397-2-6

**Published:** 2006-03-28

**Authors:** Anne-Sophie Chauvin, Alexandre Alexakis

**Affiliations:** 1 École Polytechnique Fédérale de Lausanne, LCSL, BCH 1405, CH-1015 Lausanne, Switzerland; 2 Department of Organic Chemistry, University of Geneva, 30, quai Ernest Ansermet, CH-1211 Geneva 4, Switzerland

## Abstract

The use of organophosphorus derivatising agents, prepared from C_2_ symmetric trimethylsilylated diamines, for the ^1^H NMR and ^31^P NMR determination of the enantiomeric composition of chiral alcohols is described.

## Introduction

NMR spectroscopy is one of the most frequently employed methods used for determining the enantiomeric purity of chiral compounds, based on the formation of diastereomeric complexes or derivatives [1]. Among these methods, ^31^P is a very attractive nucleus to be used for NMR analysis because of the large chemical dispersion and the simplicity of the spectra [2]. Some of the chiral phosphorous chemical derivatisation agents (CDAs) developed contain an amine or a *C*_2_ symmetric diamine moiety, and have conveniently been applied to the determination of the enantiomeric excess (ee) of various chiral alcohols, [3–20], amines,[6,13–14,21–22] thiols[3,10,12–13,22–23] and amino acids[21,24–25]. Analyses are also sometimes made by ^1^H or ^19^F NMR [26]. This is the case with α-methoxy-α-(trifluoromethyl)phenylacetic acid (Mosher's, MTPA) esters[27] and other arylmethoxyacetic acid derivatives for the determination of enantiomeric excesses and attribution of configuration of certain alcohols, amines and carboxylic acids. The scope and limitations of these methods have been discussed by Seco et al [28]. However, the analysis of the ^1^H NMR spectra is often more complicated than that of ^31^P NMR spectra because of the H-H coupling, the lower resolution and the superposition of the desired signals with other proton signals. We therefore decided to develop new CDAs to determine the enantiomeric composition by a simple ^1^H NMR method.

**Scheme 1:**
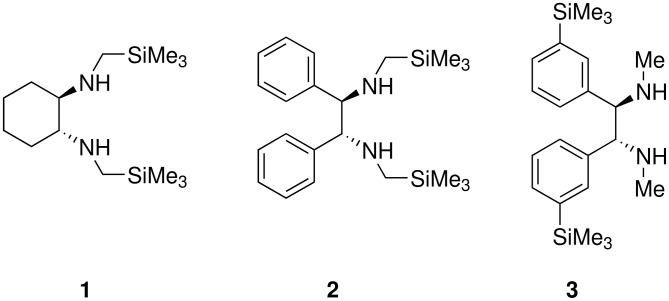
C_2_-diamines used in this study.

To be a convenient method, the signal to be observed should belong to an area usually devoid of any other signals, which can allow the determination of ee by integration. The area around 0 ppm usually fulfils these criteria. We already reported the use of TADDOL phosphorus derivatives, where the determination of ee can be performed by integration of the TADDOL isopropylidene-methyl signals [29]. Herein we report the use of CDAs **1**, **2** and **3**, obtained from *C*_2_ symmetric trimethylsilylated diamines (Scheme 1), for the determination of the enantiomeric composition of chiral alcohols, including phenylcarbinols, not only by ^31^P NMR but also by ^1^H NMR. The choice of the position of the TMS group was essential, to see if it is more discriminating on the nitrogen atom or on the backbone.

## Results and discussion

**Scheme 2:**
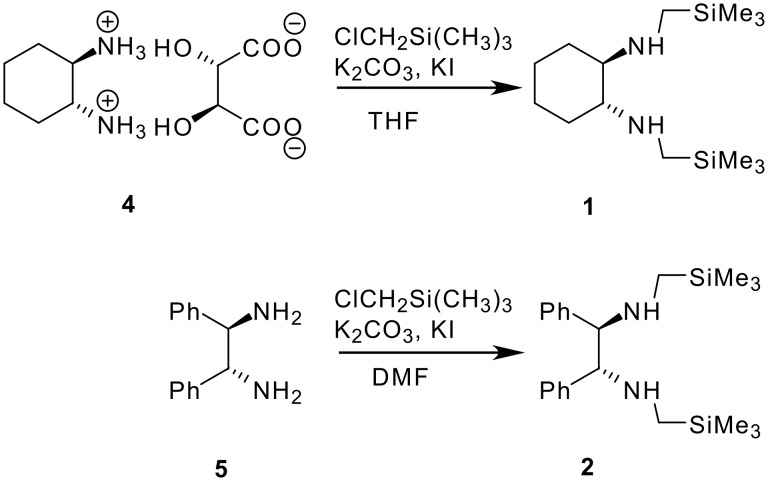
Synthesis of diamines **1** and **2**.

**Scheme 3:**
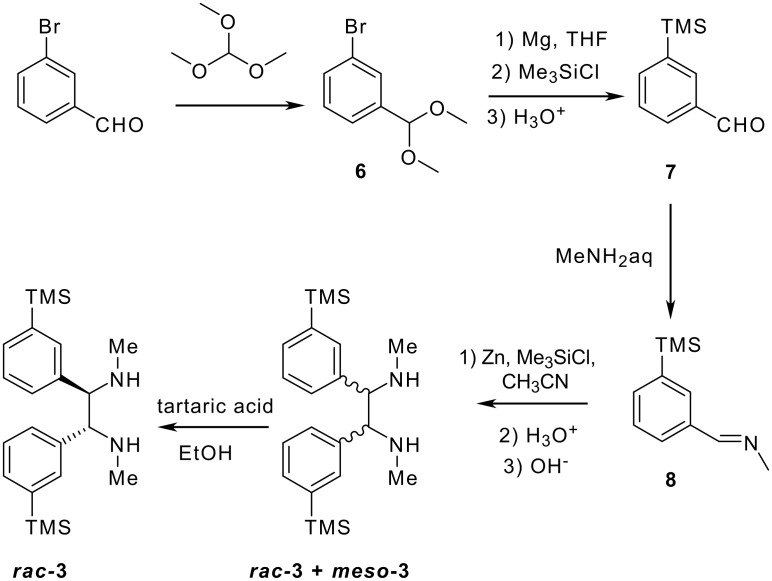
Synthesis of diamine **3**.

(*R,R*)-*N,N'*-Bis-trimethylsilanylmethylcyclohexane-1,2-diamine **1** was synthesised starting from the stable (*R,R*)-1,2-diammoniumcyclohexane mono-(+)-tartrate salt **4** and chloromethyltrimethylsilane (Scheme 2), according to a described procedure [30]. (*R,R*)-1,2-diphenyl-*N,N'*-bis-trimethylsilanylmethyl-ethane-1,2-diamine **2** was prepared by the same method, starting from (*R,R*)-diphenyl-ethane-1,2-diamine **5** (Scheme 2) [31]. *N,N'*-Dimethyl-1,2-bis-(3-trimethylsilanyl-phenyl)-ethane-1,2-diamine **3** was synthesised by reductive coupling of methyl-(3-trimethylsilanylbenzylidene)imine **8**, which was synthesised in a three step procedure according to Scheme 3. Only the racemic diamine was evaluated for the chemical shift difference Δδ by ^31^P and ^1^H NMR. All experimental details are given in Supporting Information File 1.

In order to increase the ^31^P chemical shift differences of P(III) derivatives in the presence of chiral alcohols, it was found that the best position for substituting the phenyl ring of the *C*_2_ symmetrical diamines is *meta* compared to *ortho* or *para* position [32]. It is for this reason that we introduced the methyltrimethylsilyl substituent in the *meta* position of diamine 3 (Scheme 3).

With butan-2-ol taken as representative example, a comparison of the ^31^P chemical shift differences Δδ (ppm) of some *C*_2_ symmetrical diamine-P(III) derivatives is presented in Table 1. The Δδ between the two diastereoisomers is of the same magnitude when the nitrogen atom is substituted by a methyl or a methyltrimethylsilyl moiety, despite the latter being a more bulky substituent (**9** and **10**, or **11** and **12**). However, the Δδ value increases by a factor of two in the case of cyclohexane-1,2-diamine derivatives (**9** and **10**) compared to 1,2-diphenyl-ethane-1,2-diamine derivatives (**11** to **13**) and can be explained by the rigidity of the cyclohexane ring. In the case of **11** and **13**, the chemical shift difference is roughly the same, indicating that the introduction of the trimethylsilyl moiety in the aromatic ring has more or less no influence. In all cases, the resolution of both diastereoisomeric peaks is large enough to allow the determination of ee by integration of their corresponding areas.

**Table 1:** ^31^P chemical shift differences Δδ (ppm) of some butan-2-ol P-(III) derivatives (in CDCl_3_).

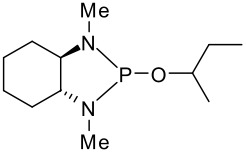	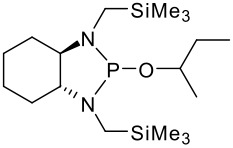	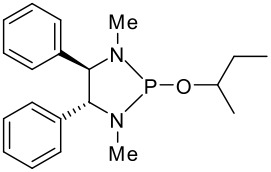	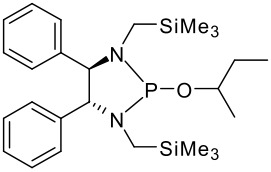	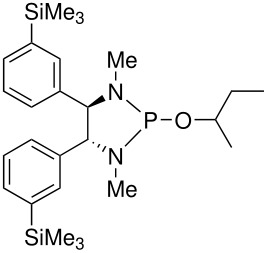
**9**	**10**	**11**	**12**	**13**
Δδ = 3.86	Δδ = 3.21	Δδ = 1.82	Δδ = 1.55	Δδ = 1.75

**Figure 1:**
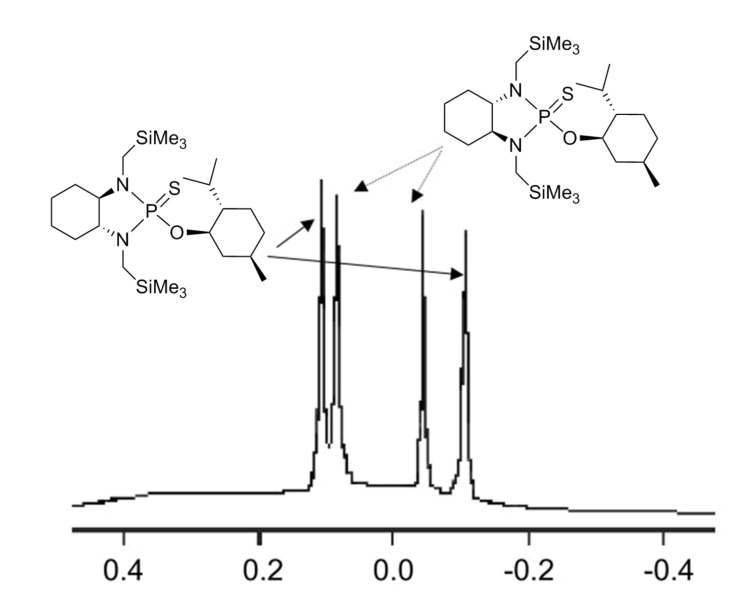
^1^H NMR spectrum in the -0.4↔0.6 ppm region of 2-(2-isopropyl-5-methyl-cyclohexyloxy)-1,3-bis-trimethylsilanylmethyl-octahydro-benzo [1,3,2] diazaphosphole 2-sulfide.

More interesting is the application of these silylated compounds for the direct determination of the ee by ^1^H NMR. The chemical shift δ (in ppm) of two diastereoisomers obtained from the same *C*_2_ symmetrical diamine-P(III) derivatives and a chiral alcohol are presented in Table 2. For each diastereoisomer, two singlets are observed in the region around 0 ppm, due to the two non-equivalent trimethysilyl substituents (see Figure 1). No other signal can interfere in this region. Futhermore, these signals are very intense, due to the presence of nine equivalent protons for the three methyls of each trimethysilyl substituent. As observed in Table 2, the resolution of the signals is good enough, so that the determination of the enantiomeric excess by integration of each diastereoisomer peak can be easily performed. This ee determination can be made either with the P(III) or with the P(V) derivatives, after reaction with sulphur, although superposition of peaks occurs more often in the latter case. With some *N,N'*-dimethyl-1,2-bis-(3-trimethylsilanyl-phenyl)-ethane-1,2-diamine **3** derivatives, especially the P(V) ones, the resolution of the two trimethylsilyl groups of one diastereoisomer decreases or does not exist, probably due to the compound's greater symmetry, but the separation between each diastereoisomer remains very good. In such a case, it is better to integrate both areas of peaks belonging to the same diastereoisomers. The ee values have been found to be the same (within experimental errors) when measured by integration of ^31^P or of ^1^H peaks (for both P(III) or P(V) derivatives). Interestingly, in the examples studied, the chemical shift difference Δδ between the two peaks of the diastereoisomer arising from the (*R,R*)-*C*_2_-diamine and the (*R*)-alcohol is often higher than that arising from the (*S,S*)-*C*_2_-diamine. This observation could on occasion be considered for the assignment of the absolute configuration of these chiral alcohols.

**Table 2:** Chemical shifts of the methyls of trimethylsilyls substituents.

	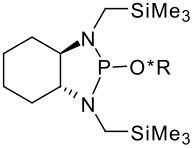		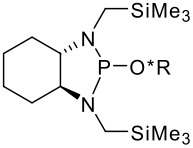		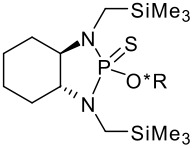		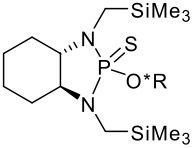	
	*(R,R)*		*(S,S)*		*(R,R)*		*(S,S)*	
	δ (ppm)		δ (ppm)		δ (ppm)		δ (ppm)	
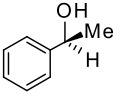	+0.12	-0.06	+0.06	-0.02	0.08	-0.10	0.11	-0.05
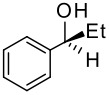	0.09	-0.03	0.07	-0.00	0.09	-0.08	0.12	-0.11
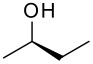	0.14	-0.05	-0.03	-0.12	0.09	-0.06	0.10	-0.09
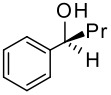	0.26	-0.06	0.12	0.10	0.10	0.06	0.08	0.11

	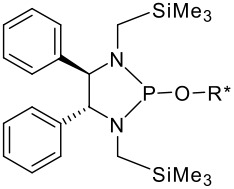		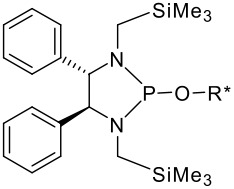		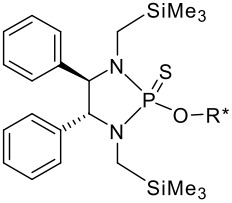		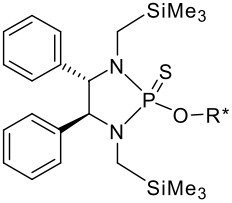	
	*(R,R)*		*(S,S)*		*(R,R)*		*(S,S)*	
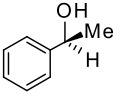	+0.11	-0.04	+0.09	-0.10	-0.04	-0.10	-0.11	-0.39
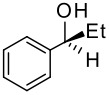	0.01	-0.06	0.08	-0.17	-0.16	-0.28	-0.11	-0.41
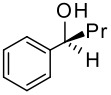	0.09	-0.07	0.10	-0.14	0.06	-0.29	-0.11	-0.39
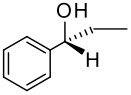	0.05	-0.03	0.06	-0.03	+0.11	-0.12	0.02	-0.03

	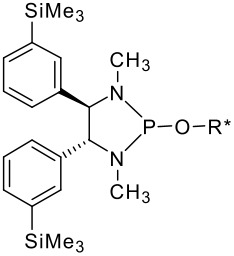		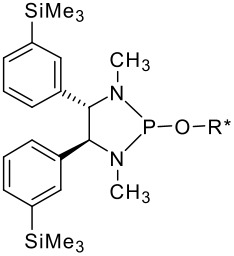		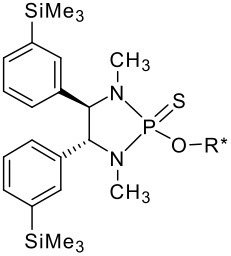		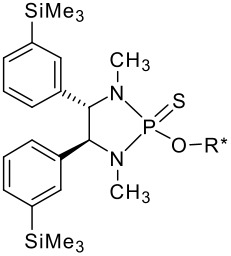	
	*(R,R)*		*(S,S)*		*(R,R)*		*(S,S)*	
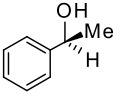	0.15	0.14	0.11	0.10	0.15	0.11	0.10	0.09
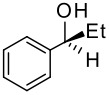	0.13	0.11	0.09	0.08	0.13	0.12	0.10	0.08
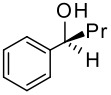	0.12	0.09	0.08	0.06	0.15	0.15	0.03	0.00
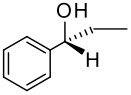	0.12	0.10	0.08	0.05	0.14	0.14	0.02	0.01

## Conclusion

In conclusion, we have synthesized three new C_2_ symmetric silylated diamines **1**, **2** and **3**, which can be easily used as CDAs for the ^1^H NMR and ^31^P NMR determination of the enantiomeric composition of secondary chiral alcohols, including phenylcarbinols. Diamines **1** and **2** are the most suitable for such a determination, whereas diamine **3** affords a lower Δδ. It seems that the position of the silyl derivative is very important: it is clear that the TMS group has to be closer to the stereocenter of the alcohol, although there is no effect due to the presence of a cyclohexyl ring instead of two aromatic substituents. If one takes into account the ease of preparation of these diamines, then, (*R,R*)-*N,N'*-bis-trimethylsilanylmethyl-cyclohexane-1,2-diamine **1** would be preferred. Work is in progress to extend this methodology to the determination of the absolute configuration of chiral alcohols, as has been done with ^31^P NMR [33].

## Supporting Information

File 1 Contains all experimental data
