# Supplementary material for: Chiral trimethylsilylated C2-symmetrical diamines as phosphorous derivatizing agents for the determination of the enantiomeric excess of chiral alcohols by 1H NMR
Source: Beilstein J Org Chem. 2006 Mar 28;2:6. doi: 10.1186/1860-5397-2-6 (PMC1483824; doi:10.1186/1860-5397-2-6)
Supplement: File 1 — Contains all experimental data [file Beilstein_J_Org_Chem-02-06-s001.doc]

Chiral trimethylsilylated *C*_2_-symmetrical diamines as phosphorous derivatizing agents for the determination of the enantiomeric excess of chiral alcohols by ^1^H NMR.

Anne-sophie Chauvin,^a^ Alexandre Alexakis ^b*^

^a^École Polytechnique Fédérale de Lausanne, LCSL, BCH 1405, CH-1015 Lausanne, Switzerland

^b^ Department of Organic Chemistry,  University of Geneva, 30, quai Ernest Ansermet, CH-1211 Geneva 4, Switzerland, Tel.: +41 (0)22 37 96522; fax: +41 (0)22 37 93215; e-mail: alexandre.alexakis@chiorg.unige.ch

**Experimental part**

**(*R,R*)-*N,N'*-Bis-trimethylsilanylmethyl-cyclohexane-1,2-diamine, 1,** was synthesized according to a procedure we recently described.[[1]](#endnote-2)

**(*R,R*)-1,2-diphenyl-*N,N'*-bis-trimethylsilanylmethyl-ethane-1,2-diamine, 2**:

A solution of (*R,R*)- 1,2-diphenyl-ethane-1,2-diamine (0.5g) in anhydrous THF (50 mL), K_2_CO_3_ (5 g) and KI (0.1g) is heated at reflux with chloromethyltrimethylsilane (1.1 eq) during 120 hours. It is then filtered to remove the salts, and the solvent is evaporated on a rotatory evaporator. The crude product is dissolved in dichloromethane (150 mL) and washed with a saturated solution of K_2_CO_3_ (100 mL). This aqueous phase is washed with dichloromethane and then with diethylether. The organic layers are dried over K_2_CO_3_, then the solids are removed by filtration and the solvents are evaporated. The product is purified by chromatography (silica gel) CH_2_Cl_2_:MeOH 100 to 97/3 % v/v (42%). NMR ^1^H (CDCl_3_, 400 MHz) : δ = 0.053 (s, 18H, -Si(CH_3_)_3_), δ = 1.6-1.8 (broad s, 2H, -NH), δ = 1.79 and 1.88 (d, 2x2H, -CH_2_-Si(CH_3_)_3_), δ = 3.47 (s, 2H, -CH-NH), δ = 7.98 and 7.13 (m, 10H, H_arom_). MS (electrospray) m/z = 385.78 (MH^+^). [α]_D_^25^ = -0.69 (c 0.03, CHCl_3_)

**1-Bromo-3-dimethoxymethyl-benzene, 6**

To a solution of 3-bromobenzaldehyde (38 g, 0.2 mol) in 55 mL of methanol is added methyl orthoformate (35.6 g, 1.2 eq) and *para*-toluenesulfonicacid (1 g); The reaction is stirred during 24 hours then the acid is neutralised by addition of sodium methoxide in methanol. The solvent is removed and the yellow residue is distilled under reduced pressure (0.6 mbar, 70°C) to give 43.9 g of the pure 1-bromo-3-dimethoxymethyl-benzene (92%). NMR ^1^H  (ppm, CDCl_3_): δ = 7.67 (s, 1H, H_arom_), 7.53 (dd, 1H, H_arom_), 7.41 (dd, 1H, H_arom_), 7.29 (dd, 1H, H_arom_), 5.41 (s, 1H, -CH), 3.37 (s, 6H, -OCH_3_). ^13^C  NMR (ppm, CDCl_3_): δ = 140.4, 131.43, 129.87, 129.74, 125.33, 122.39 (C_arom_), 101.99 (-CH), 52.58 (-OCH_3_). MS (electrospray) m/z= 231.23 [MH^+^]

**3-trimethylsilanyl-benzaldehyde, 7**

This reaction is conducted under nitrogen atmosphere. A dry three-necked 500 mL flask equipped with a reflux condenser and an addition funnel is charged with magnesium (3.8 g, 0.16 mol) and anhydrous THF (150 mL). The solution of 1-bromo-3-dimethoxymethyl-benzene (35g, 0.16 mol) in THF (100 mL) is added dropwise during 1 hour and the temperature is maintained between 30 and 40°C with a water bath. When the magnesium is totally consumed, a solution of trimethylsilyl chloride (40 mL, 2 eq) is added dropwise and the resulting solution is stirred overnight at room temperature. Then, aqueous HCl 2.5 N (200 mL) is cautiously added while cooling in an ice bath, the two phases are separated and the aqueous layer is washed with diethylether (250 mL). The organic phases are dried over potassium carbonate and the solvent is removed under vacuum to give 3-trimethylsilanyl-benzaldehyde, which is used without any further purification. NMR ^1^H  (ppm, CDCl_3_): δ = 10.09 (s, -CHO), 8.07 (s, 1H, H_arom_), 7.90 (dd, 1H, H_arom_), 7.84 (dd, 1H, H_arom_), 7.56 (dd, 1H, H_arom_), 0.35 ppm (s, 9H, Si–(CH_3_)_3_). ^13^C  NMR (ppm, CDCl_3_): δ = 192.75 (-CHO), 141.81, 139.31, 135.53, 134.59, 130.55, 128.30 (C_arom_), - 1.32 (Si–(CH_3_)_3_). MS (electrospray) m/z= 179.45 [MH^+^]

**Methyl-(3-trimethylsilanyl-benzylidene)-amine, 8**

The resulting 3-trimethylsilanyl-benzaldehyde is poured into aqueous solution of methylamine 40% (150 mL) and stirred during 12 hours before extracting with diethylether (300 mL). The organic phase is dried over potassium carbonate and the solvent is removed under vacuum. Methyl-(3-trimethylsilanyl-benzylidene)-amine (12 g) is purified by distillation under reduced pressure (0.4 mbar, 70°C). NMR ^1^H  (ppm, CDCl_3_): δ = 8.36 (s, 1H, -CHN), 8.07 (s, 1H, H_arom_), 7.93 (s, 1H, H_arom_), 7.73 (s, 1H, H_arom_), 7.46 (s, 1H, H_arom_), 3.58 (s, 3H, -NCH_3_), 0.35 (s, 9H, Si–(CH_3_)_3_). ^13^C  NMR (ppm, CDCl_3_): δ = 162.82 (-CN-), 140.96, 135.48, 135.34, 132.64, 128.44, 127.93 (C_arom_), 42.29 5 (-NCH_3_), - 1.29 (Si–(CH_3_)_3_). MS (electrospray) m/z= 192.32 [MH^+^]

***N,N'*-Dimethyl-1,2-bis-(3-trimethylsilanyl-phenyl)-ethane-1,2-diamine, 3,** A three necked round bottomed flask (100 ml), is charged with Zn dust (325 mesh, 4.4 g, 68 mmol) and anhydrous acetonitrile (50 mL) under a nitrogen atmosphere. The zinc is activated by the addition of dibromo-1,2-ethane (0.5 mL) and the mixture is brought to reflux for 5 min., then allowed to cool to room temperature. At that stage, a small amount of trimethylsilylchloride (1 mL) is added, whereupon an evolution of ethylene gas is observed. The mixture is stirred for one hour and then methyl-(3-trimethylsilanyl-benzylidene)-amine (12 g, 68 mmol) dissolved in acetonitrile (70 mL) is added (10 min). Pure TMSCl (13 mL, 12 mmol) is slowly added (20 min). The temperature increased as to maintain the internal temperature below 40°C. After 1h, the mixture is cooled to 0°C and cautiously (very exothermic at the beginning) hydrolyzed with a mixture of aqueous NH_4_OH (40 mL) and of saturated aqueous solution of NH_4_Cl (100 mL). The excess Zn is then removed by filtration, the organic layer is separated and the aqueous phase washed once with Et_2_O (150 mL) and twice with CH_2_Cl_2_ (2x100 mL). The combined organic extracts are dried over K_2_CO_3_. The salts are removed by filtration, washed with Et_2_O (50 ml) and the solvents removed on a rotatory evaporator to afford a semi-solid residue. This crude diamine is dissolved in absolute EtOH (150mL), racemic tartaric acid (34 mmol, 5.0 g) is added, and the heterogeneous mixture is brought to reflux for 40 min. After cooling to room temperature, the precipitate is collected by filtration and washed twice with EtOH (2x100 mL). This salt is poured to a mixture of 35% NaOH (30 mL), water (150 mL) and Et_2_O (200 mL). After stirring for 30 min., the layers are separated, the aqueous phase extracted twice with Et_2_O (2x100 mL), and the combined organic phases are dried over K_2_CO_3_. The solvents are evaporated to provide the pure *d,l* diamine (49%, 11.8g). ^1^H  NMR (ppm, CDCl_3_): δ = 7.24 (dd, 2H, H_arom_), 7.16 (dd, 2H, H_arom_), 7.06 (dd, 2H, H_arom_), 6.93 (dd, 2H, H_arom_), 3.47 (s, 2H, -CHN), 2.29 (s, 3H, -NHCH_3_), 0.015 (s, 18H, -SiMe_3_).1.03 MS (electrospray) m/z= 385.55 [MH^+^] Anal. Calc. for C_22_H_36_N_2_Si_2_: C  68,69; H  9.43; N  7.28; Found : C  67.96; H  9.45; N  7.23%.

**Procedure for the preparation of the phosphorous CDAs:** The reaction between the chiral diamine and the alcohol was performed in an NMR tube by adding under argon one equivalent of diamine (0.1 mmol) in solution in CDCl_3_, with 5 equivalent of a base (N,N-diethylaniline or pyridine) and one equivalent of PCl_3_ followed by the chiral alcohol (0.1 mmol), as already described with other phosphorus derivatives.[[2]](#endnote-3)-[[3]](#endnote-4) The reaction occurred instantaneously, such that the ^31^P and ^1^H NMR spectra could be immediately recorded. Sulfur may be added directly to the NMR tube and the spectra were recorded again after shaking the tube, without any further purification.

1. Alexakis, A., Chauvin, A.-S., Stouvenel, R., Vrancken, E., Mutti, S., Mangeney., P. : *Tetrahedron Asymmetry,* 2001, **12**: 1171. [↑](#endnote-ref-2)
2. Alexakis, A.; Aujard, I.; Mangeney, P. : *Synlett* ,1998, **8**: 875 [↑](#endnote-ref-3)
3. Alexakis, A.; Aujard, I.; Kanger, T.; Mangeney, P. : *Organic Syntheses*, 1998, **76**: 23 [↑](#endnote-ref-4)
